# Subnormothermic acellular machine perfusion for prolonged preservation of human kidneys

**DOI:** 10.1093/bjs/znaf147

**Published:** 2025-07-23

**Authors:** Sara Deffrennes, Serena MacMillan, Anna Paterson, Michael L Nicholson, Sarah A Hosgood

**Affiliations:** Department of Development and Regeneration, Katholieke Universiteit Leuven, Leuven, Belgium; Department of Nephrology, Dialysis and Renal Transplantation, University Hospitals Leuven, Leuven, Belgium; Department of Surgery, University of Cambridge, Addenbrooke's Hospital, Cambridge, UK; Department of Histopathology, Cambridge University Hospitals NHS Foundation Trust, Cambridge, UK; Department of Surgery, University of Cambridge, Addenbrooke's Hospital, Cambridge, UK; Department of Surgery, University of Cambridge, Addenbrooke's Hospital, Cambridge, UK

Dear Editor,

The current standard of care for kidney preservation is hypothermic preservation. Nonetheless, this does not allow a safe extension of preservation time due to cold-related adenosine 5′-triphosphate (ATP) depletion and accumulation of metabolic waste products^1^. Perfusion at near-normothermic temperature can avoid cold ischaemic injury and restore cellular metabolism^2,3^. Prolonging preservation by maintaining metabolic activity may increase the donor organ pool by improving organ assessment, matching, and allocation, and allowing organ modification and repair. We investigated whether subnormothermic acellular perfusion (SNAP) at 32°C could support cellular metabolism for up to 24 h in human kidneys.

Following static cold storage (SCS), 21 human kidneys declined for transplantation were preserved for 6 h, 12 h or 24 h using SNAP (*Fig. S1*). In the 24 h group, kidneys were perfused with either urine replacement (UR) or urine recirculation (URC) (*Fig. S1*). SNAP was performed using adapted paediatric cardiopulmonary bypass technology with an acellular human serum albumin (HSA) solution. As a control, three additional kidneys were intentionally preserved for 41 h with only SCS to match the total preservation time of the 24 h SNAP group (17 h SCS + 24 h SNAP = 41 h; *Fig. S1*). After preservation, all kidneys underwent *ex vivo* reperfusion with a red-cell-based solution at 37°C to simulate post-transplant revascularization and compare the four experimental groups (*Fig. S1*).

The three SNAP cohorts were well matched for donor demographics and ischaemic times (*Table S1*). Throughout SNAP, all kidneys maintained stable perfusion parameters and similar levels of oxygen consumption (*Fig. 1a*, *Table S2*). Levels of injury markers remained stable or decreased up to 12 h of perfusion and marginally increased from 12 to 24 h of perfusion (*Fig. 1b*). The 24 h SNAP subgroup analysis comparing URC and UR showed similar results (*Fig. S2*).

**Fig. 1:**
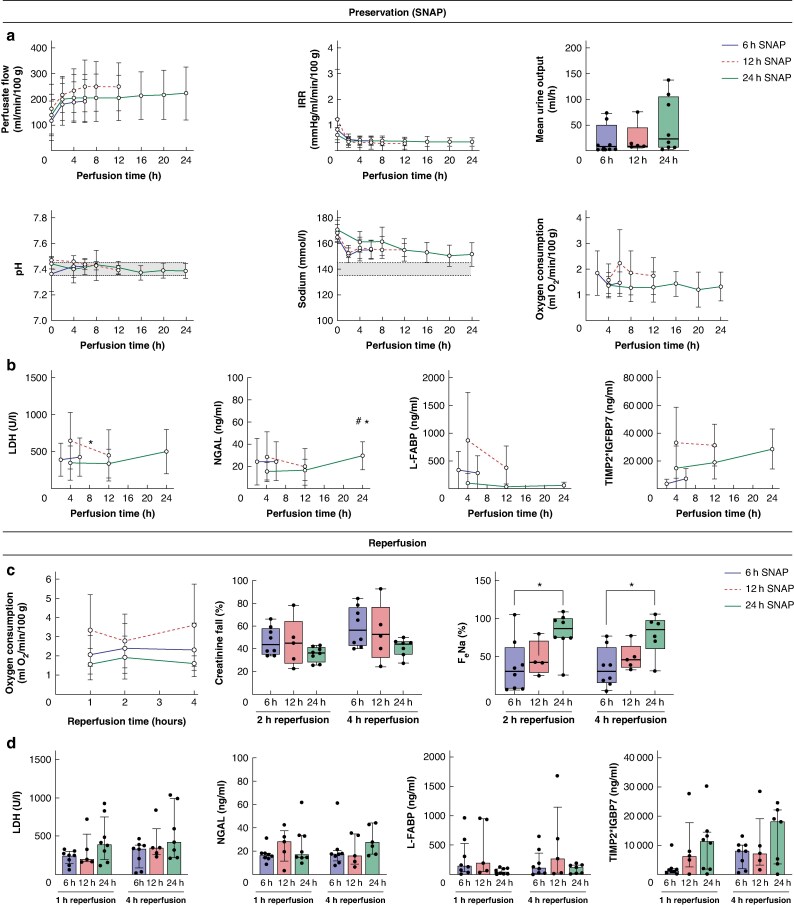
Comparison of 6 h, 12 h or 24 h of subnormothermic acellular machine perfusion (SNAP) for preservation of human kidneys

**a** Perfusate flow, intrarenal resistance (IRR), urine output, pH, sodium levels and oxygen consumption in kidneys perfused for 6 h (blue), 12 h (red) or 24 h (green) of SNAP. **b** Time course of injury markers lactate dehydrogenase (LDH) (*P < 0.05 12 h perfusion timepoint *versus* 4 h perfusion timepoint in 12h SNAP group, paired two-tailed Student *t*-test), neutrophil gelatinase-associated lipocalin (NGAL) (*P < 0.05 24 h perfusion timepoint *versus* 4 h perfusion timepoint in 24h SNAP group; ^#^*P* < 0.05 24 h perfusion timepoint *versus* 12 h perfusion timepoint in 24h SNAP group; repeated measures ANOVA), liver-type fatty acid-binding protein (L-FABP), and tissue inhibitor of metalloproteinases 2 (TIMP2)*insulin-like growth factor binding protein 7 (IGFBP7) in kidneys perfused for 6 h, 12 h or 24 h of SNAP. **c** Comparison of oxygen consumption, creatinine fall and fractional sodium excretion (F_e_Na) (*P < 0.05, one-way ANOVA) during reperfusion of kidneys perfused for 6 h, 12 h or 24 h of SNAP. **d** Injury markers LDH, NGAL, L-FABP, and TIMP2*IGFBP7 at 1 and 4 h of reperfusion of kidneys perfused for 6 h, 12 h or 24 h of SNAP. In XY graphs and bar plots, error bars show mean ± standard deviation. For boxplots, the central mark indicates the median; bottom and top edges indicate the 25th over 75th percentiles; the whiskers extend to the most extreme data points.

At reperfusion, all kidneys demonstrated functionality with oxygen consumption, sodium reabsorption and creatinine clearance (*Fig. 1c*, *Fig. S3*). There was evidence of additional tubular dysfunction in the 24 h SNAP kidneys (*Fig. 1c,d*). Histological assessment demonstrated a preserved renal morphology in all kidneys (*Fig. S4*), with no significant difference regarding injury between the groups at the end of SNAP (*P* = 0.2973) and after reperfusion (*P* = 0.5453) (*Tables S4, S5*). However, the 24 h SNAP kidneys had a numerically higher injury score (*Table S4*).

Compared to kidneys preserved for 24 h with SNAP, SCS kidneys showed poor functionality during reperfusion with significantly lower renal blood flow, reduced creatinine clearance and numerically lower urine output (*Fig. S5a*). They also released higher levels of injury markers (*Fig. S5b*) and sustained significantly more histological damage (*P* = 0.0152) (*Fig. S4*, *Tables S4, S5*).

To our knowledge, this is the first report of extended kidney preservation at 32°C with an acellular perfusate. All kidneys showed functionality and metabolic activity with adequate levels of oxygen consumption during perfusion and reperfusion. In the 24 h SNAP group, there were signs of mild deterioration after 12 h with increased levels of injury markers and histological change during reperfusion. Further investigation is needed to determine if refining the nutrient requirements or perfusate would further support viability^4^.

Compared to previous reports of (near-)normothermic machine perfusion, the absence of red blood cells in the perfusate represents a significant advantage by reducing cost, simplifying the procedure, eliminating the risk of disease transmission and avoiding the harmful effects of haemolysis. Further, as the perfusate contains Ringer’s lactate and HSA solution as principal components with no animal-derived supplements it can be translated clinically. A comparable perfusate was recently used to perfuse porcine kidneys for 24 h at 22–25°C followed by successful autotransplantation^5^.

We demonstrate that SNAP enables functional and metabolic preservation of human kidneys for up to 24 h following SCS, providing a clinically feasible strategy to extend preservation time during transplantation.

## Supplementary Material

znaf147_Supplementary_Data

## Data Availability

The data that support the findings of this study are available in the Supplementary material of this article. Additional information is available from the corresponding author upon reasonable request.
