# Supplementary material for: Subnormothermic acellular machine perfusion for prolonged preservation of human kidneys
Source: Br J Surg. 2025 Jul 23;112(7):znaf147. doi: 10.1093/bjs/znaf147 (PMC12284878; doi:10.1093/bjs/znaf147)
Supplement: znaf147_Supplementary_Data [file znaf147_supplementary_data.docx]

**Subnormothermic acellular machine perfusion for prolonged preservation of human kidneys**

Sara Deffrennes MD^1,2^, Serena MacMillan PhD^3^, Anna Paterson PhD^4^, Michael L. Nicholson ScD^3^, Sarah A. Hosgood PhD^3^

^1^Department of Development and Regeneration, Katholieke Universiteit Leuven, Leuven, Belgium

^2^Department of Nephrology, Dialysis and Renal Transplantation, University Hospitals Leuven, Leuven, Belgium

^3^Department of Surgery, University of Cambridge, Addenbrooke's Hospital, Cambridge, UK

^4^Department of Histopathology, Cambridge University Hospitals NHS Foundation Trust, Cambridge, UK

Corresponding author: Dr Sarah Hosgood, [sh744@cam.ac.uk](mailto:sh744@cam.ac.uk)

**Supplementary Material – Index**

| **Supplementary Methods** |  |
| --- | --- |
| Study population | *page 3* |
| Subnormothermic acellular machine perfusion (SNAP) | *page 3* |
| Reperfusion ex vivo | *page 4* |
| Calculated measurements | *page 5* |
| Biochemical analysis and injury markers | *page 6* |
| Haematoxylin and eosin (H&E) staining and histological analysis | *page 7* |
| Statistical analyses | *page 7* |
| **Supplemental Appendices** |  |
| Results | *page 8* |
| Discussion | *page 9* |
| **Supplementary Figures and Tables** |  |
| Figure S1 | *page 11* |
| Figure S2 | *page 12* |
| Figure S3 | *page 13* |
| Figure S4 | *page 14* |
| Figure S5 | *page 15* |
| Table S1 | *page 16* |
| Table S2 | *page 17* |
| Table S3 | *page 18* |
| Table S4 | *page 19* |
| Table S5 | *page 20* |
| **References** | *page 21* |

1. **Supplementary methods**

***Study population***

Twenty-four human kidneys from deceased donors and declined for transplantation were included in this study. Written consent for the use of kidneys for research was given by the donor families and was obtained by Specialist Nurses in Organ Donation. Ethical approval was obtained from NRES: 15/NE/0408 and 22/WA/0167.

***Subnormothermic acellular machine perfusion (SNAP)***

*Perfusion protocol*

Each kidney was retrieved at the donor centre following standard protocols, flushed and preserved on ice with UW solution prior to transportation to the research laboratory. Upon arrival at our centre, the kidneys were benched and flushed with cooled Ringer’s solution. Kidneys were then randomly assigned to perfusion for 6h (N = 8), 12h (N = 5) or 24h (N = 8) at 32°C using adapted paediatric cardiopulmonary bypass technology (Medtronic), as previously described.^1^ Pump speed was set at 1450 RPM with a target pressure of 75 mmHg. The kidneys were perfused with 500 ml of oxygenated acellular perfusate (composition below). Amino acid solution (Synthamin 17 10%) was constantly infused at a rate of 10 ml/h. Glucose 5% infusion rate was set at 3 ml/h at the start of perfusion and subsequently titrated to achieve perfusate glucose concentrations of approximately 3 mg/dL. The oxygenator was oxygenated with a gas mixture of 95%O_2_/5% CO_2_ at a flow rate of 0.2–0.3 L/min. Throughout perfusion, the pH was adjusted through titration with sodium bicarbonate 8.4% to the physiological level of 7.35–7.45. All kidneys perfused for 6h and 12h of SNAP and four kidneys perfused for 24h of SNAP were perfused with urine replacement (UR) where Ringer’s lactate was infused to replace the excreted urine as a 1:1 volume replenishment. The other four kidneys undergoing 24h of SNAP were perfused using urine recirculation (URC). Perfusate flow rate and mean arterial pressure (MAP) were recorded every 30 min. Perfusate and urine samples were collected at regular intervals (2h interval for 6h SNAP cohort and 4h interval for 12h and 24h SNAP cohorts), flash-frozen in liquid nitrogen and stored long-term at –80°C. Wedge biopsies were taken at the specified time points and were formalin-fixed and paraffin-embedded (FFPE).

*Perfusate composition*

Ringer’s solution 250 ml

Human serum albumin 5% 250 ml

Dexamethasone 6 mg

Calcium gluconate (10%) 5 ml

Sodium bicarbonate 8.4% 15 ml

Creatinine 0.110 g

Meropenem 500 mg

Verapamil 2.5 mg

*Supplements and infusions*

Synthamin 17 (10%) with 10 ml/h

- 5 ml multivitamins
- 15 ml sodium bicarbonate
- 100 IU insulin

Glucose 5% 3 ml/h

Glyceryltrinitrate (GTN) 1 mg/h

Ringer’s solution to replace urine output ml for ml (= urine replacement (UR))

***Reperfusion ex vivo***

*Perfusion protocol*

After SNAP, kidneys underwent ex vivo reperfusion at 37°C for 4h as a model of revascularisation. Three additional kidneys with a static cold storage (SCS) time equivalent to the total preservation time (cold ischaemia time + 24h SNAP) of the 24h SNAP group (i.e. approximately 41h) were also reperfused as a comparative control group. The reperfusion solution had similar composition to the SNAP perfusate with the addition of 1 unit of compatible packed red blood cells (RBCs). Renal blood flow (RBF) and MAP were recorded every 30 min and urine output hourly. Perfusate and urine samples were collected hourly, flash-frozen in liquid nitrogen and stored long-term at –80°C. Biopsies were taken at the specified time points.

*Perfusate composition*

Packed red blood cells (blood group O) 250 ml

Ringer’s solution 200 ml

Human serum albumin 5% 50 ml

Creatinine 0.110 g

Heparin 3000 IU

Sodium bicarbonate 8.4% 27 ml

Calcium gluconate 5 ml

***Calculated measurements***

Oxygen delivery, oxygen consumption and oxygen extraction were calculated from blood gas analysis of arterial and venous partial oxygen tension, total haemoglobin concentration and oxygen saturation in the perfusate based on Fick’s equation, taking into account the flow rate and kidney weight. Fractional sodium excretion, intrarenal resistance (IRR) and creatinine fall were calculated using the appropriate equations.

*Oxygen consumption during subnormothermic acellular perfusion (32°C)*

$$Oxygen consumption \left( VO_{2} \right)(ml/min/100g)= Q \times(CaO_{2}- CvO_{2})$$

Where:

$Q$ is flow in ml/min/100g;

$CaO_{2}$ is arterial oxygen content: $CaO_{2}= \times PaO2$

$CvO_{2}$ is venous oxygen content: $CvO_{2}= \times PvO_{2}$

$PaO2$is arterial oxygen pressure in kPa

$PvO_{2}$ is venous oxygen pressure in kPa

κ = solubility coefficient (0.023)

$$Oxygen delivery \left( DO_{2} \right)(ml/min)= Q \times CaO_{2}$$

$$Oxygen extraction (OER)= VO_{2}/DO_{2}$$

*Oxygen consumption during reperfusion with red blood cells (37°C)*

$$Oxygen consumption \left( VO_{2} \right)(ml/min/100g)= Q \times(CaO_{2}- CvO_{2})$$

Where:

$Q$ is flow in ml/min/100g

$CaO_{2}$ is arterial oxygen content: $CaO_{2}=\left( \times PaO2 \right)+(1.34 \times Hb\times SaO_{2})$

$CvO_{2}$ is venous oxygen content: $CvO_{2}=\left( \times PvO_{2} \right)+\left( 1.34\times Hb\times SvO_{2} \right)$

Hb = haemoglobin (mg/dL)

α = haemoglobin binding capacity (1.34)

κ = solubility coefficient (0.021)

*Fractional sodium excretion (F_e_Na)*

$$FeNa \left( \% \right)=\frac{Na \left( urine \right)\times Creatinine \left( perfusate \right)}{Na \left( perfusate \right)\times Creatinine \left( urine \right)}\times100$$

$$Intrarenal resistance (IRR) (mmHg/ml/min)=\frac{MAP}{perfusate flow}$$

Where:

MAP = mean arterial pressure

*Creatinine fall*

$$Creatinine fall (\%)=\frac{Creatinine \left( T_{1} \right)-Creatinine \left( T_{2} \right)}{Creatinine (T_{1})} \times100$$

Where:

$Creatinine \left( T_{1} \right)$ is creatinine concentration at timepoint 1

$Creatinine \left( T_{2} \right)$ is creatinine concentration at timepoint 2

***Biochemical analysis* *and injury markers***

Fresh arterial perfusate and urine samples taken at regular intervals were sent to the NHS Clinical Biochemistry facility at Addenbrooke’s Hospital for measurements of serum creatinine, urine creatinine and electrolytes.

Levels of neutrophil gelatinase-associated lipocalin (NGAL), an early acute kidney injury (AKI) marker released by damaged distal tubules, liver-type fatty acid-binding protein 1 (L-FABP), a very early marker of AKI released by proximal tubular cells, tissue inhibitors of metalloproteinases 2 (TIMP-2) and insulin-like growth factor binding protein related protein 7 (IGFBP-7), two cell cycle arrest markers indicating cellular stress, were measured in the urine samples at the specified timepoints by a quantitative sandwich enzyme immunoassay technique using Duoset ELISA kits (R&D Systems Minneapolis, MN) according to the manufacturer’s instructions. Results were obtained using a FLUOstar Optima plate reader (BMG Labtech, Ortenberg, Germany).

Lactate dehydrogenase (LDH) activity, a general marker of cellular damage, was measured in perfusate samples at the specified timepoints using the LDH Activity Assay kit (Sigma Aldrich, Saint Louis, MO, USA). Results were obtained using a FLUOstar Optima plate reader (BMG Labtech, Ortenberg, Germany).

***Haematoxylin and eosin (H&E) staining and histological analysis***

H&E staining was completed by the Human Research Tissue Bank at Addenbrooke’s Hospital on 4 μm FFPE sections. All H&E-stained sections were imaged using Axioscan 7 (Zeiss, Jena, Germany) and analysed using QuPath (v0.5.0). Prior to scoring, all images were anonymised. A consultant renal pathologist provided an injury report blinded to the experimental groups and according to the criteria outlined in Supplementary Table 3.

***Statistical analyses***

Continuous data were tested for normality with the Shapiro–Wilk test. Average values are presented as mean ± standard deviation (SD) for data following a normal distribution and median with interquartile range (IQR) if otherwise. Comparisons between two groups were performed using the two-tailed Student’s *t*-test or the Wilcoxon rank-sum test. Comparisons between three or more groups were performed using ANOVA or Kruskal–Wallis tests with Bonferroni or Dunn’s multiple comparisons correction. Comparisons within a group at two different timepoints were performed with a paired *t*-test or a Wilcoxon matched-pairs signed rank test. Comparisons within a group at three different timepoints were performed with a repeated measures ANOVA or Friedman test. Two-sided *p*-values of ≤ 0.050 were considered statistically significant. GraphPad Prism® 10 (GraphPad software, California, USA) was used for statistical analysis.

1. **Supplementary Appendices**

***Results***

*Comparison of 24h SNAP with urine recirculation (URC) vs urine replacement (UR)*

In the 24h SNAP subgroup analysis based on urine replacement strategy (URC vs UR), no difference in mean sodium perfusate levels was observed between URC and UR (*p* = 0.8913) (Fig. S2a). The mean pH in the 24h SNAP kidneys with URC (pH 7.36 ± 0.03) was significantly lower than in 24h SNAP kidneys with UR (pH 7.45 ± 0.05; *p* = 0.0271), although levels in both groups were within physiological range (Fig. S2a). There was no significant difference in bicarbonate supplementation during SNAP between both groups (*p* = 0.3590) (Fig. S2a).

With regard to the injury markers, we observed different dynamics for NGAL and [TIMP-2] × [IGFBP7] in the 24h SNAP with URC compared to UR groups (Fig. S2b). Levels of these injury markers were lower at 4h perfusion in the kidneys perfused with URC but then continuously increased throughout SNAP, reflecting a constant release and recirculation of these injury markers. For 24h SNAP kidneys with UR, levels decreased from 4h to 12h of perfusion and then increased numerically from 12h to 24h (Fig. S2b).

*Assessment of kidney function during ex vivo reperfusion after 6h, 12h and 24h of SNAP*

Upon reperfusion, RBF gradually increased in the first hour and stabilized thereafter (Fig. S3). The area under the curve (AUC) for RBF was not significantly different between the three groups (*p* = 0.573) (Fig. S3). The kidneys maintained stable oxygen consumption with levels similar to those during SNAP (Fig. S3). Levels were numerically higher in the 12h SNAP kidneys but there was no significant difference between the groups (*p* = 0.0709). There was no significant difference in the level of urine output between the groups (*p* = 0.2531) (Fig. 1c). A decrement in perfusate creatinine was observed in all kidneys (Fig. 1c). Fractional sodium excretion (F_e_Na) was significantly higher in the 24h SNAP kidneys compared to 6h SNAP kidneys at 2h of reperfusion (*p* = 0.0244) and 4h of reperfusion (*p* = 0.0133) and numerically higher in the 24h SNAP kidneys compared to the 12h SNAP kidneys (Fig. 1c).

There were no significant differences in the absolute levels of the injury markers LDH, NGAL, L-FABP and [TIMP-2] × [IGFBP7] among the three SNAP groups, although the levels of [TIMP-2] × [IGFBP7] were numerically higher in the 24h SNAP kidneys (*p* = 0.3538) (Fig. 1d).

***Discussion***

This study demonstrated the successful perfusion of human kidneys for up to 24h at the subnormothermic temperature of 32°C using an acellular perfusate. SNAP enabled the total preservation period to exceed 40h when combining the warm ischaemia time (WIT), cold ischaemia time (CIT) and SNAP duration (Table S1).

During reperfusion, all kidneys preserved with SNAP demonstrated organ functionality and viability. This was supported by the histological findings showing preserved renal morphology with mild to moderate tubular injury after 24h of SNAP. In contrast, kidneys preserved by SCS for an equivalent preservation period to the 24h SNAP kidneys showed a high level of injury with poor function during reperfusion.

To our knowledge, this is the first report to propose the use of an acellular perfusate to preserve kidneys at a viable temperature of 32°C. Although it might seem counter-intuitive that kidneys can be perfused without the addition of a specific oxygen carrier, the efficiency of modern membrane oxygenators are so high that supra-physiological perfusate PO_2_ can be achieved.

The absence of RBCs in the perfusate represents a significant advantage by reducing cost, simplifying the procedure, eliminating the risk of disease transmission from third parties and avoiding the harmful effects of haemolysis. In the past, synthetic oxygen carriers such as those based on bovine haemoglobin or perfluorocarbon have been proposed as a substitute for RBCs during normothermic machine perfusion (NMP); however, adverse effects and safety concerns have been reported, which has prevented their clinical application.^2–4^ Studies investigating NMP (at 37°C) without the addition of an oxygen carrier show conflicting results.^5–7^ We therefore opted for a small reduction in perfusion temperature to 32°C, thereby reducing the oxygen requirements of the kidney but still maintaining a high level of metabolism. Our previous work in porcine kidneys showed that this was a viable approach and it has been demonstrated by others that at a temperature of 28°C there is sufficient metabolic activity to support cellular repair processes after warm ischaemia.^8,9^

Another important advantage of the study is that the perfusate can be clinically translated. It contains Ringer’s lactate and human serum albumin solution as principal components with no addition of animal-derived supplements. A comparable perfusate was recently used to perfuse porcine kidneys for 24h at room temperature (22–25°C) using a donation after circulatory death (DCD) model. Kidneys were then autotransplanted with improved function compared to SCS kidneys.^10^ Tissue culture medium-based perfusates for prolonged kidney perfusion have also been investigated. de Haan et al. reported the perfusion of human kidneys at 25°C for 4 days with a perfusate containing Dulbecco's Modified Eagle Medium/F12 and human serum albumin solution.^11^ Although function and metabolism were evident after 4 days in transplant-declined human kidneys, when subsequently transplanted in a porcine model, kidneys were not able to sustain life.

Although perfusion and reperfusion were successful in all kidneys preserved with SNAP, in the 24h SNAP group there were signs of mild deterioration after 12h with increased levels of injury markers, histological change and significantly higher F_e_Na. Further investigation is needed to determine if additional nutrient requirements or modifications to the perfusate are needed to support viability after 12h of SNAP. Nonetheless, we cannot rule out that the pre-existing ischaemic injury and donor factors are contributing factors.

Urine recirculation was previously proposed as an alternative to urine replacement to achieve more stable haemodynamics and homeostasis during prolonged NMP.^12,13^ In our study, urine recirculation applied during 24h of SNAP did not show a clear advantage over urine replacement with regard to haemodynamic parameters and electrolyte and acid–base homeostasis, although the numbers were small. We note that although urine production was variable, it tended to be much lower than we and others have previously reported.^13,14^ Therefore, we can speculate that the advantage of urine recirculation is only apparent when large volumes of urine are produced and replenished with crystalloids, causing electrolyte imbalance.

Although the kidneys included in the study were declined for transplantation they were of good quality with all donors having normal kidney function at the time of retrieval. Nonetheless, the study has some limitations. First, single human kidneys from different deceased donors rather than pairs were included in the study. To account for this variability the minimum group size for the SNAP cohorts was N = 5. Importantly, we found no significant differences in donor demographics, kidney weight, WIT and CIT between the experimental SNAP groups and therefore the groups were comparable. Second, the study included a short 4h reperfusion phase as a means to assess viability. A transplant model would be needed to determine longer-term graft function. Nonetheless, we are confident that the 4h reperfusion phase is sufficient to test the metabolic behaviour of kidneys after the different durations of SNAP.^15^ Third, we restricted our comparison of SNAP with SCS to determine the feasibility of SNAP. A comparison of other modes of preservation were beyond the remit of this study but should be the focus of future research.

In summary, this study demonstrates that human kidneys can be successfully perfused with SNAP for up to 24h to extend the preservation period to a total preservation time of up to 41h. The presented perfusion set-up provides a clinically feasible strategy to extend preservation time in transplantation.

1. **Supplementary Figures and Tables**

**
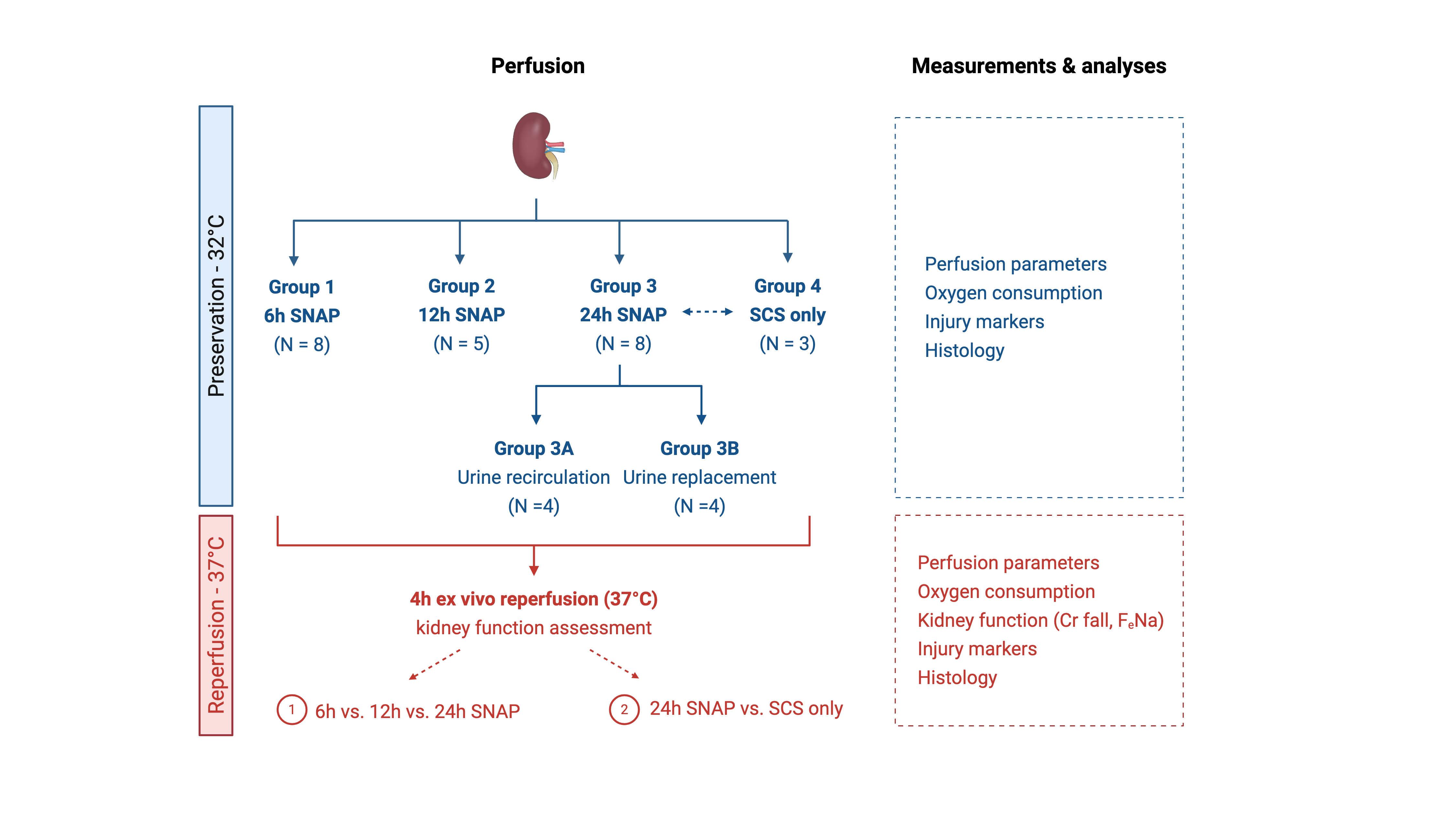
**

**Figure S1: Experimental set-up.** Twenty-four (N = 24) human kidneys were included in the study. After retrieval, the kidneys were transported to the laboratory on ice (i.e. static cold storage (SCS)). Subsequently, eight kidneys underwent 6h of subnormothermic (32°C) acellular perfusion (SNAP), five kidneys 12h of SNAP and eight kidneys 24h of SNAP. Four of the 24h SNAP kidneys were perfused with urine replacement (UR) and four kidneys were perfused with urine recirculation (URC). Additionally, three kidneys were preserved with SCS for 41h to match the total preservation time of the 24h SNAP group (17h SCS + 24h SNAP = 41h). This enabled comparison of 24h of SNAP preservation with SCS preservation. After preservation, all 24 kidneys underwent 4h of ex vivo reperfusion at 37°C to simulate post-transplant revascularisation and compare kidney function between the four experimental groups.

**
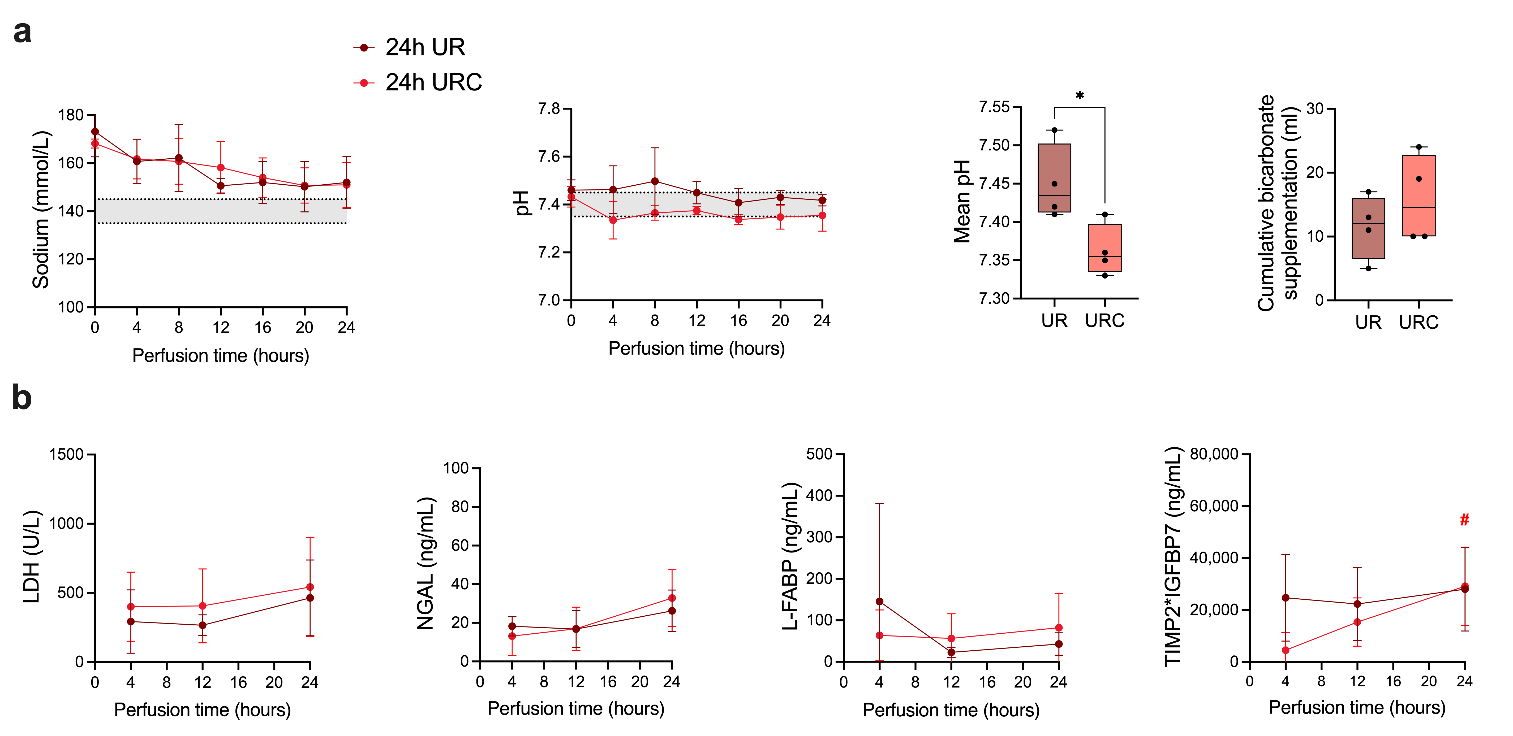
**

**Figure S2: Comparison of 24h SNAP with urine recirculation (URC) vs urine replacement (UR). (a)** Sodium levels, pH (**P* <0.05, unpaired two-tailed Student *t*-test), and cumulative bicarbonate supplementation during 24h SNAP with URC or UR; **(b)** time course of the injury markers LDH, NGAL, L-FABP and TIMP2*IGFBP7 (URC: ^#^*P* <0.05 24h perfusion timepoint vs 4h perfusion timepoint; Friedman test with Dunn’s multiple comparisons correction). In XY graphs, error bars show mean ± standard deviation. For boxplots, the central mark indicates the median; bottom and top edges indicate the 25th over 75th percentiles; the whiskers extend to the most extreme data points.

**
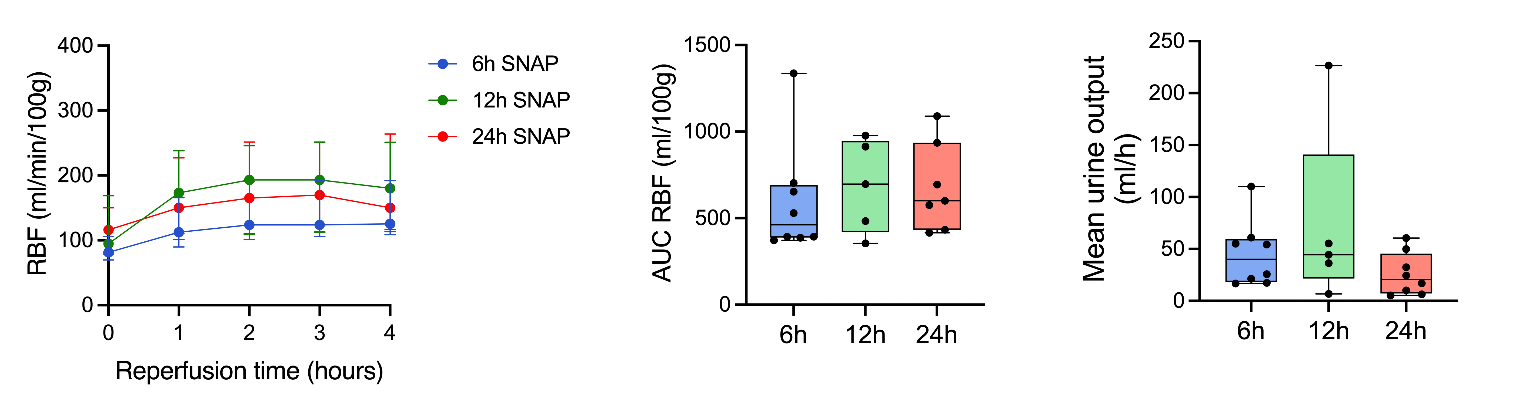
**

**Figure S3: Comparison of the 6h, 12h and 24h SNAP kidneys during ex vivo reperfusion.** Renal blood flow (RBF), area under the curve (AUC) for RBF and mean urine output during ex vivo reperfusion of the kidneys preserved with 6h, 12h or 24h of SNAP. In XY graphs, error bars show mean ± standard deviation. For boxplots, the central mark indicates the median; bottom and top edges indicate the 25th over 75th percentiles; the whiskers extend to the most extreme data points.

**Figure S4: Histological assessment of kidneys preserved with 6h, 12h or 24h SNAP or with SCS only.** Representative images of haematoxylin and eosin (H&E)-stained kidney cortical biopsies prior to perfusion (pre), after SNAP and after reperfusion in the 6h, 12h, 24h SNAP and SCS group. Scale bar: 100 μm.

**
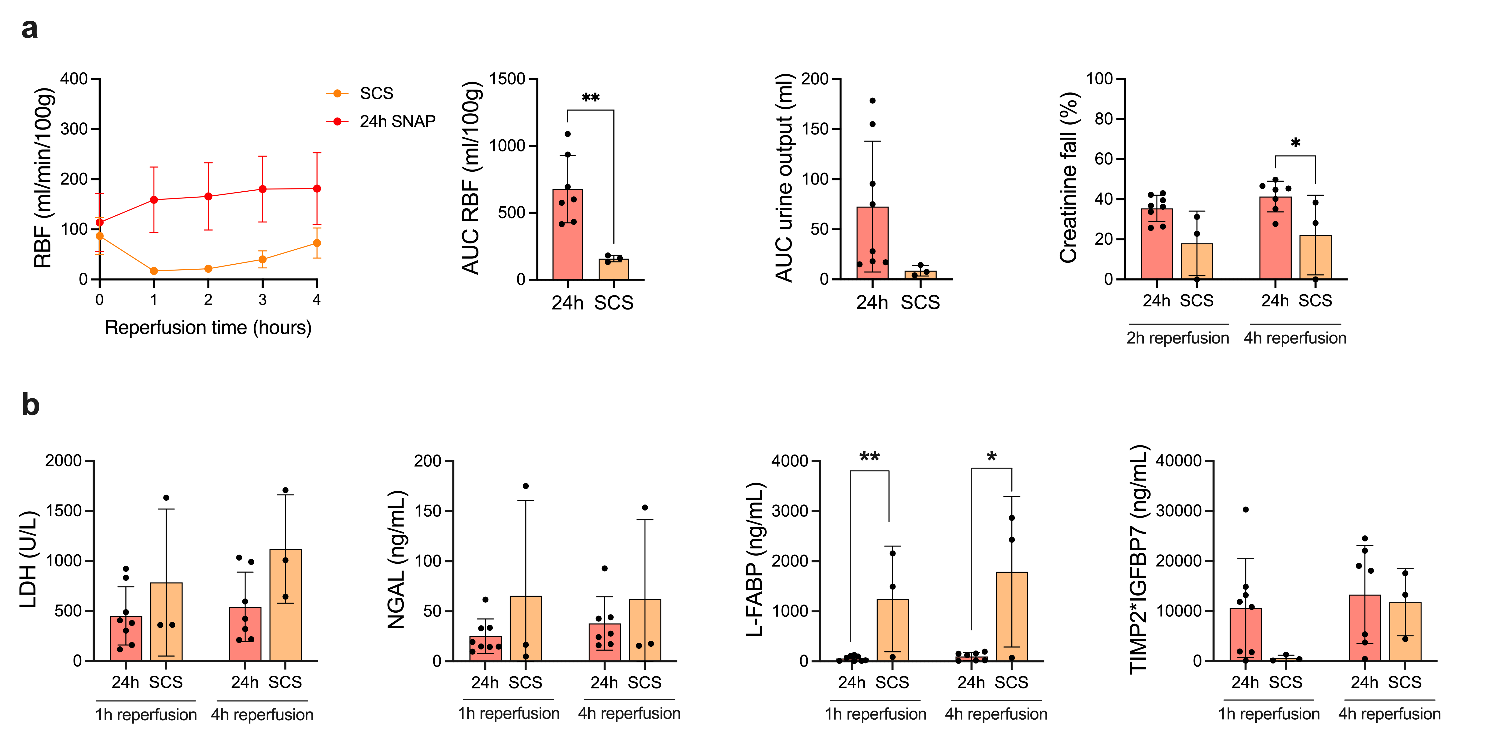
**

**Figure S5: Comparison of the 24h SNAP and SCS kidneys during ex vivo reperfusion. (a)** Renal blood flow (RBF), area under the curve (AUC) for RBF (^**^*P* < 0.01, unpaired two-tailed Student *t*-test), AUC for urine output and creatinine fall (^*^*P* < 0.05, unpaired two-tailed Student *t*-test) during reperfusion of the kidneys preserved with 24h SNAP vs SCS. **(b)** Levels of injury markers LDH, NGAL, L-FABP (^*^*P* < 0.05, ^**^*P <* 0.01, unpaired two-tailed Student *t*-test) and TIMP2*IGFBP7 at 1h and 4h reperfusion of kidneys preserved with 24h SNAP vs SCS. In XY graphs, error bars show mean ± standard deviation. For bar plots, error bars show mean ± standard deviation.

**Table S1: Donor demographics for the kidneys in the 6h SNAP, 12h SNAP, 24h SNAP and SCS cohorts**

|  | **6h cohort (N = 8)** | **12h cohort (N = 5)** | **24h cohort (N = 8)** | **SCS (N = 3)** | ***P* value** |
| --- | --- | --- | --- | --- | --- |
| **Donor age (median; IQR)** | 70 (60–74) | 67 (56–69) | 72 (62–77) | 61 (37–79) | *p* = 0.6705 |
| **Male** | 5 | 2 | 5 | 1 |  |
| **Female** | 3 | 3 | 3 | 2 |  |
| **DBD** | 5 | 1 | 3 | 0 |  |
| **DCD** | 3 | 4 | 5 | 3 |  |
| **Terminal creatinine (μmol/l)** | 70 (±25) | 78 (±33) | 73 (±25) | 64 (±12) | *p* = 0.9059 |
| **WIT (min) (median; IQR)** | 12 (12–14) | 13 (11–16) | 16 (15–19) | 12 (10–13) | *p* = 0.0608 |
| **CIT (min)** | 1309 (± 605) | 1156 (± 301) | 1029 (± 316) | 2366 (± 90) |  |
| **Total preservation time (= WIT + CIT + SNAP) (min)** | 1719 (± 570) | 1887 (± 3295) | 2500 (± 344) | 2377 (± 89) | SCS vs 24h *p* = 0.5689 |
| **Kidney weight (g)** | 205 (±63) | 224 (±117) | 200 (±50) | 189 (±79) | *p* = 0.9154 |
| **Cause of death** | | | | | |
| Intracranial haemorrhage | 4 | 4 | 4 | 2 |  |
| Hypoxic brain injury | 3 |  | 3 |  |  |
| Ischaemic heart disease | 1 |  | 1 |  |  |
| Brain tumour |  |  |  | 1 |  |
| **Reason for discard** | | | | | |
| PMH or age |  |  | 3 | 1 |  |
| No capacity at hospital |  |  | 1 |  |  |
| CIT | 1 |  | 1 |  |  |
| (Suspected) malignancy | 1 | 2 |  |  |  |
| Anatomy | 5 | 1 | 2 | 2 |  |
| Poor perfusion |  |  | 1 |  |  |
| Remuzzi score | 1 | 1 |  |  |  |
| No suitable recipient |  | 1 |  |  |  |

*N* value refers to number of kidneys in each group. Summary values are mean ± standard deviation unless otherwise described in the table. For comparison of donor age and warm ischaemia time (WIT) between groups, a Kruskal–Wallis test was completed with Dunn’s multiple comparisons correction. For comparison of cold ischaemia time (CIT), terminal serum creatinine and kidney weight, a one-way ANOVA was completed with Bonferroni’s multiple comparisons correction. Total preservation time was compared between 24h SNAP and SCS with an unpaired two-tailed Student *t*-test. *P*-values are shown to four decimal places. DBD: donation after brain death; DCD: donation after circulatory death; CIT: cold ischaemia time: WIT: warm ischaemia time; PMH: past medical history.

**Table S2: Summary of respiratory parameters during 6h, 12h and 24h of SNAP**

|  | **6h cohort (N = 8)** | **12h cohort (N = 5)** | **24h cohort (N = 8)** | ***P* value** |
| --- | --- | --- | --- | --- |
| **Oxygen consumption (ml/min/100g)** | | | | |
| Minimum | 0.98 | 0.36 | 0.50 |  |
| Maximum | 3.49 | 3.63 | 2.38 |  |
| Mean (±SD) | 1.50 (±0.4) | 1.88 (±0.86) | 1.27 (±0.47) | *p* = 0.1975 |
| **Oxygen extraction (%)** | | | | |
| Minimum | 24 | 22 | 8 |  |
| Maximum | 68 | 62 | 71 |  |
| Mean (±SD) | 45 (±10) | 40 (±9) | 38 (±18) | *p* = 0.5722 |

*N* value refers to number of kidneys in each group. Minimum, maximum and mean values for oxygen consumption and extraction within each group are specified. To compare oxygen consumption and extraction between the groups, a one-way ANOVA was completed with Bonferroni’s multiple comparisons correction. *P*-values are shown to four decimal places.

**Table S3: Histological scoring system for assessing acute glomerular and tubular injury in cortical wedge biopsies**

|  | **Percentage of field affected** | | | |
| --- | --- | --- | --- | --- |
|  | **0%** | **0**–**30%** | **30**–**60%** | **>60%** |
| **Tubular dilation** | 0 | 1 | 2 | 3 |
| **Tubular debris** | 0 | 1 | 2 | 3 |
| **Tubular necrosis** | 0 | 1 | 2 | 3 |
| **Vacuolation** | 0 | 1 | 2 | 3 |
| **Interstitial edema** | 0 | 1 | 2 | 3 |
| **Glomerular thrombi (clumping)** | 0 | 1 | 2 | 3 |
| **Glomerular shrinkage** | 0 | 1 | 2 | 3 |
| **Cumulative score** | /21 | | | |

**Table S4: Comparison of injury score in cortical wedge biopsies between 6h, 12h, 24h of SNAP and SCS**

| **Timepoint** | **Injury score** | | | |
| --- | --- | --- | --- | --- |
|  | **SCS cohort**  **(N = 3)** | **6h SNAP cohort**  **(N = 8)** | **12h SNAP cohort**  **(N = 5)** | **24h SNAP cohort**  **(N = 8)** |
| Prior to perfusion | 3.0 (± 1.7) | 7.6 (± 1.8) | 6.0 (± 4.2) | 5.6 (± 1.9) |
| End of SNAP | – | 6.9 (± 2.5) | 7.8 (± 3.4) | 9.0 (± 2.3) |
| End of ex vivo reperfusion | 10.7 (± 4.9) | 7.9 (± 3.1) | 7.4 (± 2.6) | 8.1 (± 2.9) |
| △ end of reperfusion – prior perfusion | 7.7 (± 4.5)^#^ | 0.3 (± 2.5) | 1.4 (± 4.8) | 2.5 (± 1.6) |
| ^#^SCS vs 24 SNAP (*p =* 0.0152) | | | | |

*N* value refers to number of kidneys in each group. Summary values are mean ± standard deviation. Data were compared between the SNAP groups with a one-way ANOVA with Bonferroni’s multiple comparison test. Data were compared between 24h SNAP and SCS using an unpaired two-tailed Student *t*-test*. P*-values are shown to four decimal places.

**Table S5: Injury score in cortical wedge biopsies provided per kidney for each respective timepoint**

|  | | **Injury score** | | | |
| --- | --- | --- | --- | --- | --- |
|  |  | **SCS (N = 3)** | **6h cohort (N = 8)** | **12h cohort (N = 5)** | **24h cohort (N = 8)** |
| **Kidney 1** | Prior to perfusion | 2 | 10 | 4 | 4 |
|  | End of SNAP | - | 9 | 2 | 11 |
|  | End of reperfusion | 14 | 8 | 5 | 8 |
| **Kidney 2** | Prior to perfusion | 5 | 6 | 0 | 4 |
|  | End of SNAP | - | 5 | 11 | 10 |
|  | End of reperfusion | 13 | 5 | 7 | 7 |
| **Kidney 3** | Prior to perfusion | 2 | 10 | 11 | 6 |
|  | End of SNAP | - | 9 | 9 | 7 |
|  | End of reperfusion | 5 | 14 | 5 | 7 |
| **Kidney 4** | Prior to perfusion |  | 7 | 7 | 8 |
|  | End of SNAP |  | 8 | 9 | 9 |
|  | End of reperfusion |  | 11 | 11 | 10 |
| **Kidney 5** | Prior to perfusion |  | 9 | 8 | 4 |
|  | End of SNAP |  | 5 | 8 | 6 |
|  | End of reperfusion |  | 7 | 9 | 4 |
| **Kidney 6** | Prior to perfusion |  | 6 |  | 9 |
|  | End of SNAP |  | 6 |  | 13 |
|  | End of reperfusion |  | 5 |  | 14 |
| **Kidney 7** | Prior to perfusion |  | 7 |  | 5 |
|  | End of SNAP |  | 3 |  | 8 |
|  | End of reperfusion |  | 6 |  | 8 |
| **Kidney 8** | Prior to perfusion |  | 6 |  | 5 |
|  | End of SNAP |  | 10 |  | 8 |
|  | End of reperfusion |  | 7 |  | 7 |

**References**

1. Nicholson, M.L. and Hosgood, S.A. Renal transplantation after ex vivo normothermic perfusion: the first clinical study. *Am J Transplant* **13**, 1246–1252 (2013).

2. Arykbaeva, A.S.*, et al.* Red blood cells as oxygen carrier during normothermic machine perfusion of kidney grafts: Friend or foe? *Am J Transplant* (2024).

3. Kim, J.-H., Jung, E.-A. and Kim, J.-E. Perfluorocarbon-based artificial oxygen carriers for red blood cell substitutes: considerations and direction of technology. *Journal of Pharmaceutical Investigation* **54**, 267–282 (2024).

4. Edgworth, E.*, et al.* HBOC-301 in Porcine Kidney Normothermic Machine Perfusion and the Effect of Vitamin C on Methemoglobin Formation. *Antioxidants (Basel)* **11**(2022).

5. Pool, M.B.F.*, et al.* Ex-Vivo Kidney Perfusion With Hemoglobin-Based Oxygen Carriers, Red Blood Cells, or No Oxygen Carrier. *J Surg Res* **301**, 248–258 (2024).

6. von Horn, C.*, et al.* The impact of oxygen supply and erythrocytes during normothermic kidney perfusion. *Sci Rep* **13**, 2021 (2023).

7. Longchamp, A.*, et al.* Acellular Perfusate is an Adequate Alternative to Packed Red Blood Cells During Normothermic Human Kidney Perfusion. *Transplant Direct* **10**, e1609 (2024).

8. Adams, T.D., Patel, M., Hosgood, S.A. and Nicholson, M.L. Lowering Perfusate Temperature From 37°C to 32°C Diminishes Function in a Porcine Model of Ex Vivo Kidney Perfusion. *Transplant Direct* **3**, e140 (2017).

9. Andrijevic, D.*, et al.* Cellular recovery after prolonged warm ischaemia of the whole body. *Nature* **608**, 405–412 (2022).

10. Abraham, N.*, et al.* Subnormothermic Oxygenated Machine Perfusion (24 h) in DCD Kidney Transplantation. *Transplant Direct* **10**, e1633 (2024).

11. de Haan, M.J.A.*, et al.* A cell-free nutrient-supplemented perfusate allows four-day ex vivo metabolic preservation of human kidneys. *Nat Commun* **15**, 3818 (2024).

12. Weissenbacher, A.*, et al.* Urine recirculation prolongs normothermic kidney perfusion via more optimal metabolic homeostasis-a proteomics study. *Am J Transplant* **21**, 1740–1753 (2021).

13. Weissenbacher, A.*, et al.* Urine Recirculation Improves Hemodynamics and Enhances Function in Normothermic Kidney Perfusion. *Transplant Direct* **6**, e541 (2020).

14. Hosgood, S.A., Thompson, E., Moore, T., Wilson, C.H. and Nicholson, M.L. Normothermic machine perfusion for the assessment and transplantation of declined human kidneys from donation after circulatory death donors. *Br J Surg* **105**, 388–394 (2018).

15. De Beule, J., De Craemer, S., Verstraeten, L., Ghesquiere, B. and Jochmans, I. Ischemia-Induced Metabolic Patterns Associate With Kidney Function During Normothermic Kidney Perfusion, a Preclinical Study. *Ann Surg* (2023).
